# Angioimmunoblastic T-Cell Lymphoma: A Case Report

**DOI:** 10.7759/cureus.25526

**Published:** 2022-05-31

**Authors:** Brittany Miles, Eseosa A Bazuaye-Ekwuyasi, Jayati Mallick, Quan D Nguyen

**Affiliations:** 1 Medical Education, University of Texas Medical Branch, Galveston, USA; 2 Radiology, University of Texas Medical Branch, Galveston, USA; 3 Diagnostic Radiology, University of Texas Medical Branch, Galveston, USA; 4 Pathology, University of Texas Medical Branch, Galveston, USA; 5 Radiology, Baylor College of Medicine, Houston, USA

**Keywords:** ipi (international prognostic index), stem cell transplant for hematological malignancies, extranodal involvement, epstein-barr virus positive diffuse large b-cell lymphoma, angioimmunoblastic t cell lymphoma

## Abstract

Previously believed to be an exaggerated immune response and not a lymphoma, angioimmunoblastic T-cell lymphoma (AITL) is now recognized as a rare variant of peripheral T-cell lymphoma with an aggressive clinical course and poor response to current therapies. There is no standard of care for treatment, but the identification of extranodal involvement is useful for prognostic purposes since the involvement of more than one extranodal site can escalate the patient’s risk category on the International Prognostic Index (IPI). Here we present the case of a patient with AITL who initially presented with an extranodal disease in the form of a fluorodeoxyglucose (FDG)-avid subcutaneous nodule and probable involvement of the spleen. After two months of treatment, her lymphoma exhibited an escalation of grade and an extensive worsening of Epstein-Barr virus (EBV) positivity.

## Introduction

Angioimmunoblastic T-Cell Lymphoma (AITL) is an aggressive peripheral T-cell lymphoma with a poor prognosis [1]. First described as a distinct entity in the 1970s, it was initially believed to represent an aggressive immune response and was not recognized as a lymphoma variant [2]. It is a rare condition, with approximately 0.05 cases per 100,000 people in the United States [1]. Most patients present with Intermediate or High-Risk disease according to the International Prognostic Index (IPI), with high-risk patients having a five-year survival rate of approximately 20% [3]. The presence of extranodal involvement can be important, as it represents a key component of the IPI scoring system [4]. Epstein Barr virus (EBV) also plays an interesting and significant role in the pathogenesis of AITL, with EBV-positive B-cell immunoblasts present in 80-90% of cases despite the neoplastic T-cells being negative for the Epstein-Barr encoding region (EBER) [5]. The EBV-positive B immunoblastic proliferation can progress and give rise to EBV-positive large B-cell lymphoma (WHO, 2016), as seen in our case. There is no standard of care for the treatment of AITL, although lower-risk patients may be offered treatment with high-dose steroids while patients with the higher-risk disease are often treated with an anthracycline-based combination regimen such as CHOP (cyclophosphamide, doxorubicin, vincristine, prednisone) [6]. Patients who achieve a complete response (CR) with initial therapy are often considered for autologous hematopoietic stem cell transplant (HSCT) [7,8].

## Case presentation

**Figure 1:**
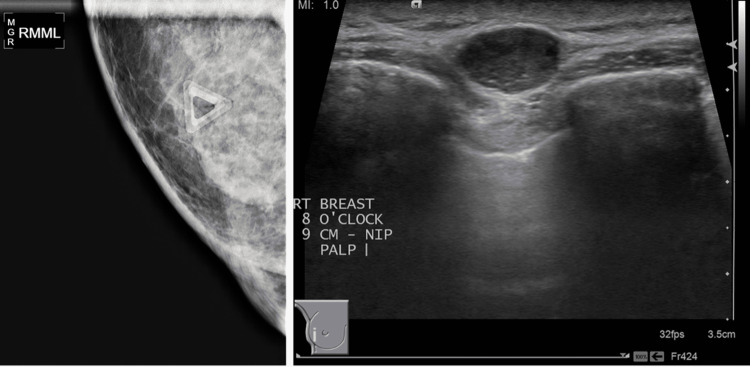
Mammogram and ultrasound findings (Left): Spot magnification medial-lateral mammogram of the right breast showing a 12 x 8 x 11 mm circumscribed lesion at the 8 o’clock location 9 cm from the nipple; (Right): Targeted right breast ultrasound showing the hypoechoic, well-circumscribed mass seen on the mammogram.

An 80-year-old patient presented with a two-year history of non-pruritic, steroid-responsive rash that primarily involved the bilateral breasts and flanks. It was biopsied on two previous occasions due to the patient’s concerns regarding possible cutaneous lymphoma. Both biopsies favored a reactive process (“age-related Epstein-Barr virus (EBV)-associated lymphoproliferative disorder”), with gene rearrangement studies showing no evidence of clonality. Shortly thereafter, a palpable 12 x 8 mm breast mass was developed, with mammogram and ultrasound findings as seen in Figure 1­­.

**Figure 2:**
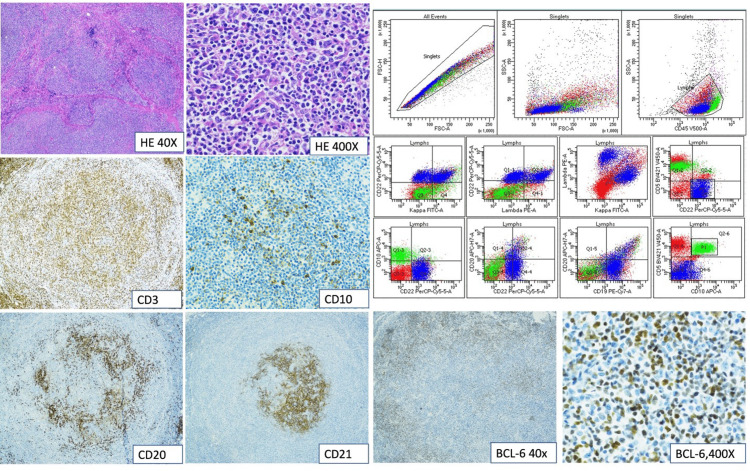
Immunohistochemical analysis and flow cytometry of the initial biopsy HE400X: Hematoxylin and eosin staining and 400X magnification; CD3, CD20, and CD30: Cellular tumor markers; EBER: Epstein-Barr encoding region

Biopsy of the breast mass showed proliferation of atypical lymphoid cells and high endothelial blood vessels in a vague nodular architecture, associated with multiple small foci of necrosis. The lymphoid cells within the nodules were comprised of both CD20, PAX-5 positive B-cells and CD3, CD2, CD4, CD5, and CD7-positive T-cells. BCL6 was positive in many of these cells, and an aberrant CD10-positive T-cell population was identified. A gene rearrangement analysis was performed but no clonality was detected (Figure 2).

**Figure 3:**
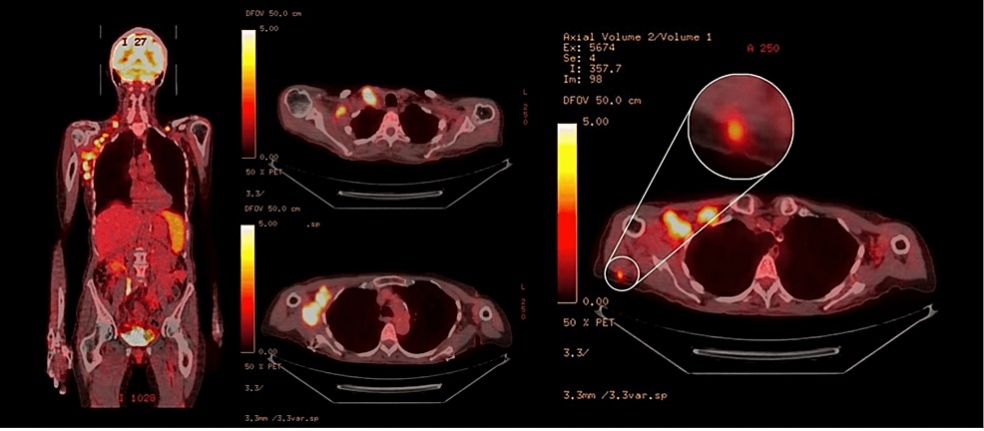
Fused images of an F-18 FDG PET-CT scan Fused images of an F-18 FDG PET-CT scan show numerous hypermetabolic lymph nodes in the right axilla. The spleen is diffusely hypermetabolic, concerning lymphomatous involvement. A hypermetabolic subcutaneous nodule in the right shoulder may also represent extranodal involvement. F-18 FDG: F-18 Fluorodeoxyglucose; PET/CT: Positron emission tomography-computed tomography

Overall, the histological, immunohistochemical, and immunophenotypic findings are consistent with angioimmunoblastic T-cell lymphoma. Her bone marrow biopsy was negative for any involvement. Positron emission tomography-computed tomography (PET/CT) showed involvement of the right neck, supraclavicular area, and axilla as well as a hypermetabolic nodule in the subcutaneous fat posterior to the right shoulder (Figure 3).

**Figure 4:**
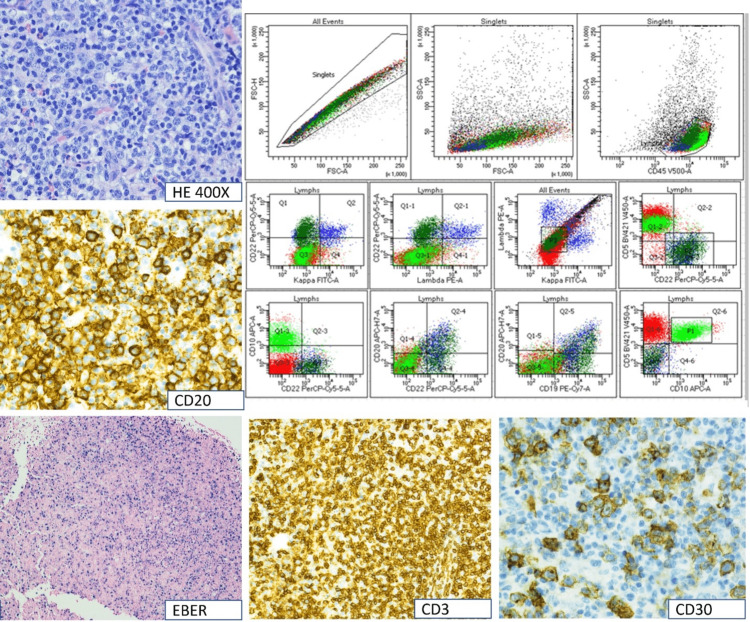
Immunohistochemical and flow cytometry results of the second biopsy.

Due to concerns about her age, she was treated with CEOP (cyclophosphamide, etoposide, vincristine, prednisone) chemotherapy (CHOP in which hydroxydaunorubicin, also known as adriamycin replaces etoposide). After an apparent initial response, significant progression developed a few months after treatment initiation, and a biopsy of a right axillary node was performed. The pathology now showed effaced lymph nodal architecture with the proliferation of large lymphoid cells with moderate cytoplasm, irregular nuclei, open chromatin, and prominent nucleoli intermingling with small lymphocytes, histiocytes, and many eosinophils. These large cells were positive for CD20 and PAX-5. These cells were also positive for CD30 and *MUM-1*. EBV in situ hybridization showed a marked increase in EBER-positive cells in comparison with the previous biopsy. Flow cytometry detected 20% aberrant CD10 positive T-cells along with 13% aberrant medium-to-large-sized B-cells without any immunoglobulin light chain expression. This aberrant B-cell population was not present in the previous flow cytometry. These findings are consistent with the emergence of an EBV-positive CD30-positive B-cell clone/EBV-positive, CD30-positive diffuse large B-cell lymphoma (DLBCL) in the background of the patient’s previously diagnosed AITL (Figure 4).

The patient’s treatment was then switched to romidepsin. After a few months of response, her disease progressed again and she was enrolled in a clinical trial with an unknown agent for which no response was seen, and a brief trial of single-agent gemcitabine was attempted. Shortly after the initiation of gemcitabine, the patient presented to the hospital with a fever and altered mental status, and the determination was made to proceed with palliative care.

## Discussion

AITL is an interesting and unusual malignancy because neoplastic EBV-negative T-cells are typically found closely associated with EBV-positive B-cells in the surrounding tumor microenvironment. DLBCL can also present concurrently with AITL at initial diagnosis or appear to develop later as an apparent evolution of the underlying malignant process. The DLBCL associated with AITL is usually EBV-positive and has commonly been regarded as a “high-grade histologic transformation” of the underlying (AITL) malignant process [9]. One study theorized that the EBV-positive malignant clone emerges as a result of the underlying immunodeficiency caused by AITL since 35% of the cases in that study developed an EBV-associated B-cell lymphoma such as DLBCL or Classical Hodgkin Lymphoma [9]. A study investigating the genetic mutations in patients with AITL determined that multiple lymphomagenic pathways appear to exist, the most common of which is associated with numerous coexistent mutations [10]. One very promising theory for the multilineage nature of this malignancy involves the development of a *TET2* mutation in an early hematopoietic progenitor cell. This would create the malignant potential for both B and T cell lines, and potentially create an environment where a malignancy in one cell line could induce deleterious effects in the other [11]. Current therapies for this malignancy are inadequate. As our understanding of AITL improves, it is hoped that new therapeutic targets will be discovered that will provide improved outcomes for what is currently a disease with a dismal prognosis.

## Conclusions

AITL is an aggressive T-cell lymphoma with a poor overall prognosis. Combination chemotherapy is typically offered to these patients, and those who respond well to initial therapy are considered for autologous stem cell transplantation. Our patient was unfortunately not a candidate for stem cell transplantation due to age limitations. She also experienced rapid progression on first-line chemotherapy due to the evolution of a de-novo EBV-positive diffuse large B-cell lymphoma that was not seen on initial presentation.
